# Supplementary material for: Abnormal and Changing Information Interaction in Adults with Attention-Deficit/Hyperactivity Disorder Based on Network Motifs
Source: Brain Sci. 2023 Sep 15;13(9):1331. doi: 10.3390/brainsci13091331 (PMC10526475; doi:10.3390/brainsci13091331)
Supplement: Supplementary file 1 [file brainsci-13-01331-s001.zip › brainsci-2560437-supplementary.pdf]

Supplementary Materials for

Abnormal and changing information interaction in adults with attention-deficit/hyperactivity disorder based on network motifs

1. Supplementary tables

Table S1 Mapping between ROIs and experiential function networks in AAL template.

ROI	Regions	RSN	ROI	Regions	RSN	ROI	Regions	RSN
1	Precentral_L	DMN	31	Cingulum_Ant_L	DMN	61	Parietal_Inf_L	Attention
2	Precentral_R	DMN	32	Cingulum_Ant_R	DMN	62	Parietal_Inf_R	Attention
3	Frontal_Sup_L	DMN	33	Cingulum_Mid_L	Subcortical	63	SupraMarginal_L	Sensorimotor
4	Frontal_Sup_R	DMN	34	Cingulum_Mid_R	Subcortical	64	SupraMarginal_R	Sensorimotor
5	Frontal_Sup_Orb_L	DMN	35	Cingulum_Post_L	DMN	65	Angular_L	Attention
6	Frontal_Sup_Orb_R	DMN	36	Cingulum_Post_R	DMN	66	Angular_R	Attention
7	Frontal_Mid_L	Attention	37	Hippocampus_L	Subcortical	67	Precuneus_L	DMN
8	Frontal_Mid_R	Attention	38	Hippocampus_R	Subcortical	68	Precuneus_R	DMN
9	Frontal_Mid_Orb_L	Attention	39	ParaHippocampal_L	Subcortical	69	Paracentral_Lobule_L	Sensorimotor
10	Frontal_Mid_Orb_R	Attention	40	ParaHippocampal_R	Subcortical	70	Paracentral_Lobule_R	Sensorimotor
11	Frontal_Inf_Oper_L	Attention	41	Amygdala_L	Subcortical	71	Caudate_L	Subcortical
12	Frontal_Inf_Oper_R	Attention	42	Amygdala_R	Subcortical	72	Caudate_R	Subcortical
13	Frontal_Inf_Tri_L	Attention	43	Calcarine_L	Visual	73	Putamen_L	Subcortical
14	Frontal_Inf_Tri_R	Attention	44	Calcarine_R	Visual	74	Putamen_R	Subcortical
15	Frontal_Inf_Orb_L	Attention	45	Cuneus_L	Visual	75	Pallidum_L	Subcortical
16	Frontal_Inf_Orb_R	Attention	46	Cuneus_R	Visual	76	Pallidum_R	Subcortical
17	Rolandic_Oper_L	Sensorimotor	47	Lingual_L	Visual	77	Thalamus_L	Subcortical
18	Rolandic_Oper_R	Sensorimotor	48	Lingual_R	Visual	78	Thalamus_R	Subcortical
19	Supp_Motor_Area_L	Attention	49	Occipital_Sup_L	Visual	79	Heschl_L	Sensorimotor
20	Supp_Motor_Area_R	Sensorimotor	50	Occipital_Sup_R	Visual	80	Heschl_R	Sensorimotor
21	Olfactory_L	Subcortical	51	Occipital_Mid_L	Visual	81	Temporal_Sup_L	Sensorimotor
22	Olfactory_R	DMN	52	Occipital_Mid_R	Visual	82	Temporal_Sup_R	Sensorimotor
23	Frontal_Sup_Medial_L	DMN	53	Occipital_Inf_L	Visual	83	Temporal_Pole_Sup_L	Attention
24	Frontal_Sup_Medial_R	DMN	54	Occipital_Inf_R	Visual	84	Temporal_Pole_Sup_R	Sensorimotor
25	Frontal_Mid_Orb_L	DMN	55	Fusiform_L	Visual	85	Temporal_Mid_L	DMN

26	Frontal_Mid_Orb_R	DMN	56	Fusiform_R	Visual	86	Temporal_Mid_R	DMN
27	Rectus_L	DMN	57	Postcentral_L	Sensorimotor	87	Temporal_Pole_Mid_L	Subcortical
28	Rectus_R	DMN	58	Postcentral_R	Sensorimotor	88	Temporal_Pole_Mid_R	Subcortical
29	Insula_L	Sensorimotor	59	Parietal_Sup_L	Sensorimotor	89	Temporal_Inf_L	Attention
30	Insula_R	Sensorimotor	60	Parietal_Sup_R	Sensorimotor	90	Temporal_Inf_R	DMN

Table S2 Brain regions showing significant differences based on node roles.

Role	ROI	Name	Network	p(FDR)	ADHD(SD)	NC(SD)
DPP	1	Precentral_L	DMN	0.003	138.35(172.63)	260.12(212.59)
DPP	38	Hippocampus_R	Subcortical	0.007	197.28(218.65)	117.04(158.30)
DPP	42	Amygdala_R	Subcortical	0.012	214.90(291.60)	101.56(138.46)
DPP	67	Precuneus_L	DMN	0.03	236.78(225.62)	436.96(448.27)
PPR	13	Frontal_Inf_Tri_L	Attention	0.014	150.66(148.59)	76.66(67.66)
PPR	48	Lingual_R	Visual	0.045	107.40(73.47)	187.12(167.18)
PPR	72	Caudate_R	Subcortical	0.04	117.33(114.4)	213.56(238.34)
PPR	89	Temporal_Inf_L	Attention	0.033	117.71(103.07)	173.64(135.84)
PPO	17	Rolandic_Oper_L	Sensorimotor	0.045	113.52(88.51)	165.82(142.18)
PPO	79	Heschl_L	Sensorimotor	0.045	118.61(92.04)	176.92(137.08)

Table S3 The directional edges of node roles with significant differences between brain regions.

ROLE	ROI	Name	Link	ROI	Name	Network	P - value
DPP	1	Precentral_L	bidirection	43	Calcarine_L	DMN- Visual	0.009
DPP	1	Precentral_L	bidirection	67	Precuneus_L	DMN-DMN	0.006
DPP	1	Precentral_L	bidirection	89	Temporal_Inf_L	DMN- Attention	0.006
DPP	67	Precuneus_L	bidirection	4	Frontal_Sup_R	DMN- DMN	0.009
DPP	67	Precuneus_L	bidirection	80	Heschl_R	DMN- Sensorimotor	0.014
DPP	38	Hippocampus_R	bidirection	42	Amygdala_R	Subcortical - Subcortical	0.0001
DPP	38	Hippocampus_R	bidirection	73	Putamen_L	Subcortical - Subcortical	0.04
DPP	38	Hippocampus_R	bidirection	76	Pallidum_R	Subcortical - Subcortical	0.011
DPP	42	Amygdala_R	bidirection	40	ParaHippocampal_R	Subcortical - Subcortical	0.028
DPP	42	Amygdala_R	bidirection	73	Putamen_L	Subcortical - Subcortical	0.017
PPR	13	Frontal_Inf_Tri_L	unidirection	41	Amygdala_L	Attention - Subcortical	0.002
PPR	48	Lingual_R	unidirection	83	Temporal_Pole_Sup_L	Visual - Attention	0.011
PPR	72	Caudate_R	unidirection	14	Frontal_Inf_Tri_R	Subcortical - Attention	0.031
PPR	89	Temporal_Inf_L	unidirection	30	Insula_R	Attention - Sensorimotor	0.033
PPO	17	Rolandic_Oper_L	unidirection	33	Cingulum_Mid_L	Sensorimotor - Subcortical	0.0002
PPO	17	Rolandic_Oper_L	unidirection	34	Cingulum_Mid_R	Sensorimotor - Subcortical	0.0005
PPO	79	Heschl_L	unidirection	1	Precentral_L	Sensorimotor -DMN	0.0007
PPO	79	Heschl_L	unidirection	33	Cingulum_Mid_L	Sensorimotor - Subcortical	0.0005

Table S4 Brain regions showing variation trend among node roles.

ROI	Name	Network	NC	Role Weight	To	ADHD	Role Weight
52	Lingual_R	Visual	DPP	0.44	→	PPR	0.41
63	Precuneus_R	Sensorimotor	DPP	0.43	→	PPR	0.41
77	Caudate_L	Subcortical	DPP	0.44	→	PPO	0.43
78	Caudate_R	Subcortical	DPP	0.43	→	PPO	0.42
89	Temporal_Pole_Sup_L	Attention	DPP	0.45	→	PPR	0.41

Table S5 Brain regions showing significant correlations with ASRS scores.

DPP role-degree				PPR role-degree			
**ID**	**Network**	**r**	**P**	**ID**	**Network**	**r**	**P**
38	Subcortical	0.293	0.006	13	Attention	0.228	0.033
42	Subcortical	0.346	0.001	48	Visual	-0.206	0.043

2. Supplementary results

In order to ensure the consistency of recognition motifs in the ADHD dataset, we detected each threshold of the AAL atlas and Power atlas, respectively (threshold ranging from 0.1 to 0.4 with a partition interval of 0.05). We identified motif patterns of groups and found that when the threshold was 30%, the population proportion of all kinds of network motifs in the two groups reached the maximum and remained stable. The specific situation is shown in Supplementary Figure S1 and Figure S2.

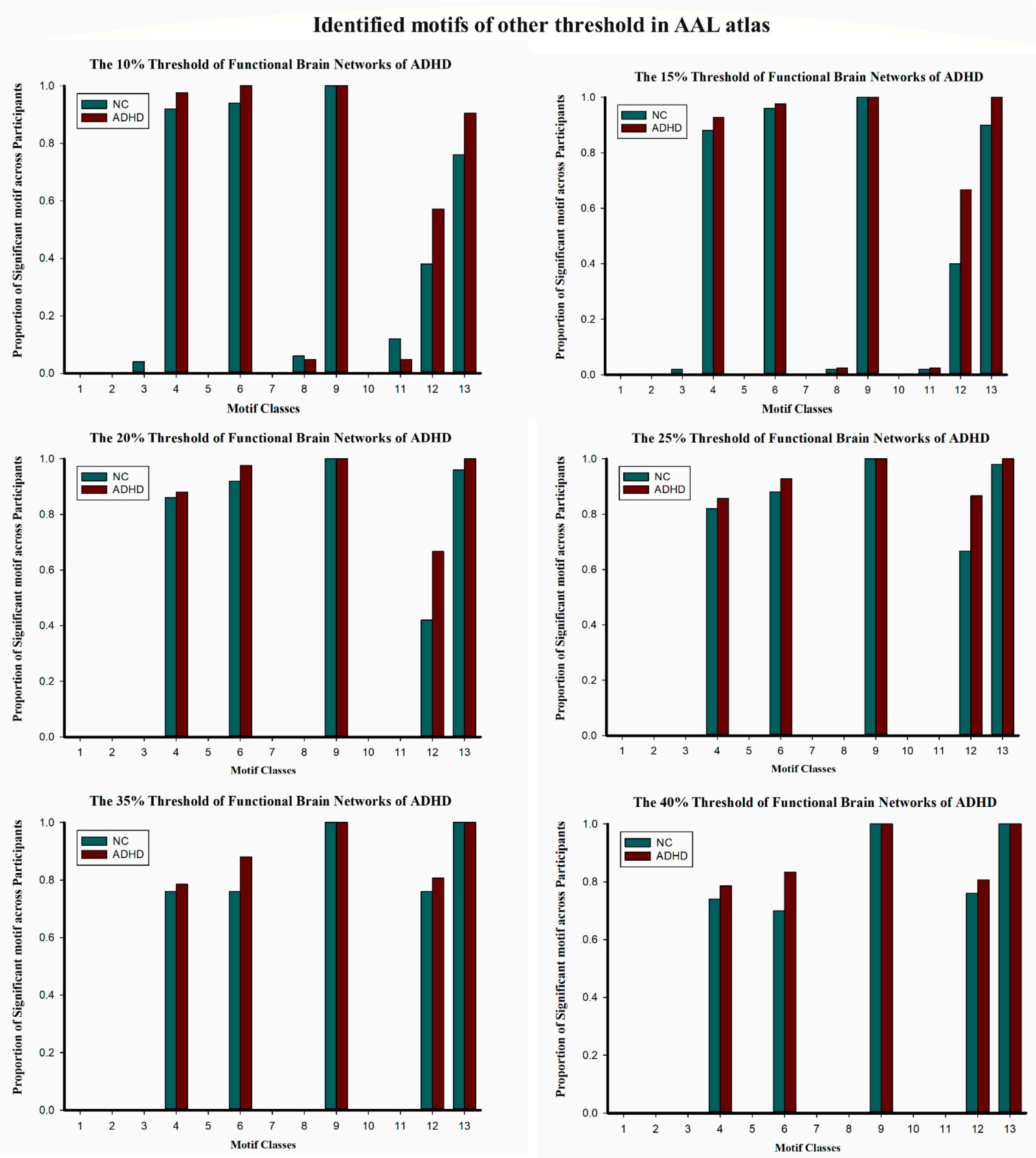

Supplementary Figure S1. The results of identified motifs in each threshold about AAL atlas.

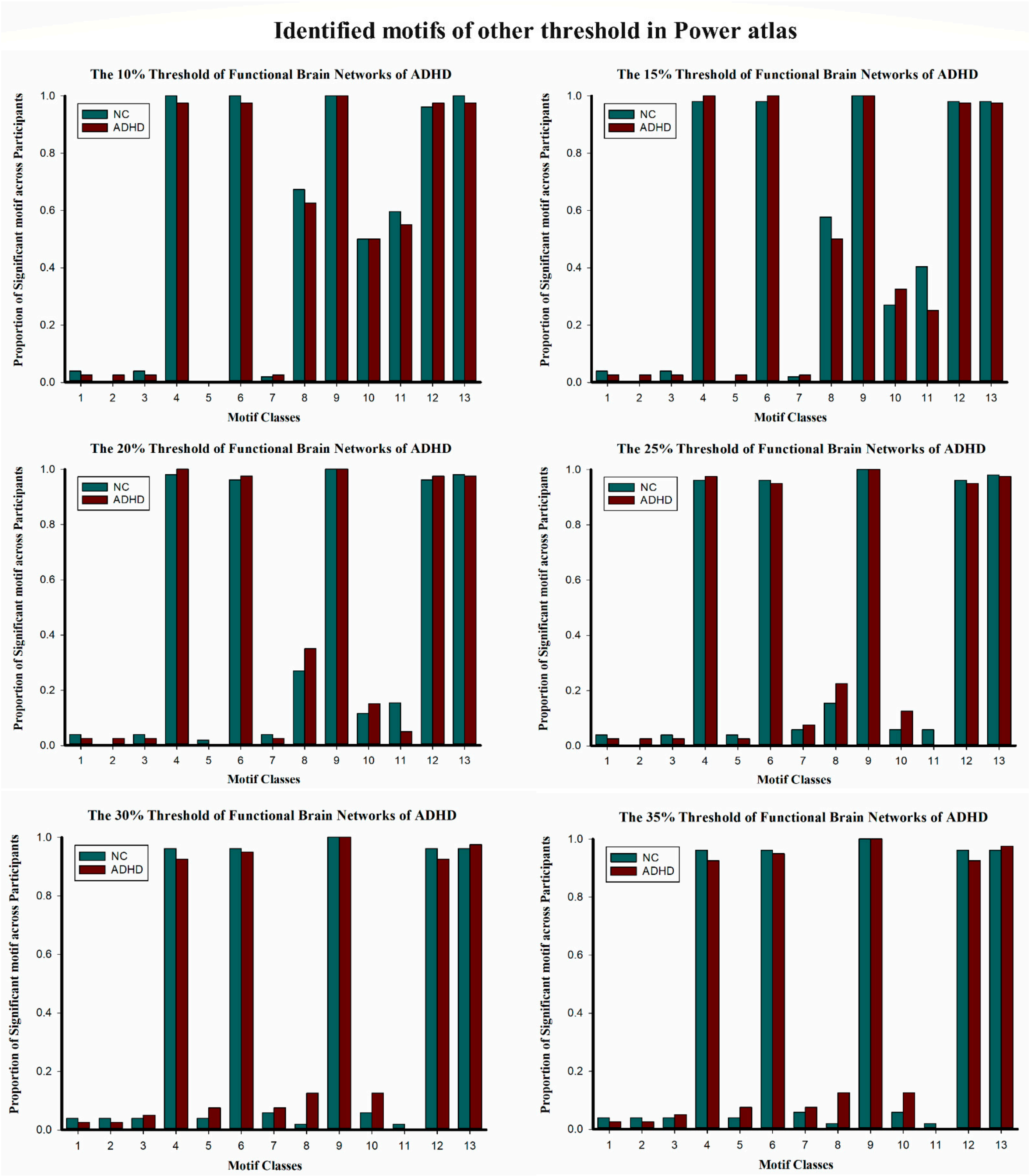

Supplementary Figure S2. The results of identified motifs in each threshold about Power atlas.

To investigate the relationship between node degree and role-degree in brain functional network, we used Pearson's correlation to measure. We found that, in two groups of subjects, DPP role is highly correlation with node total-degree, in-degree and out-degree. The PPR role is highly correlation with in-degree node (r(NC)=0.8843, r(ADHD)=0.8844, $p<0.0001$), and PPO role with out-degree node (r(NC)=0.8541, r(ADHD)=0.8569, $p<0.0001$). It indicates that each kind of node role plays a different function in information interaction. The larger the node role-degree, the stronger the information interaction ability it represents. The specific situation is shown in Supplementary Figure S3.

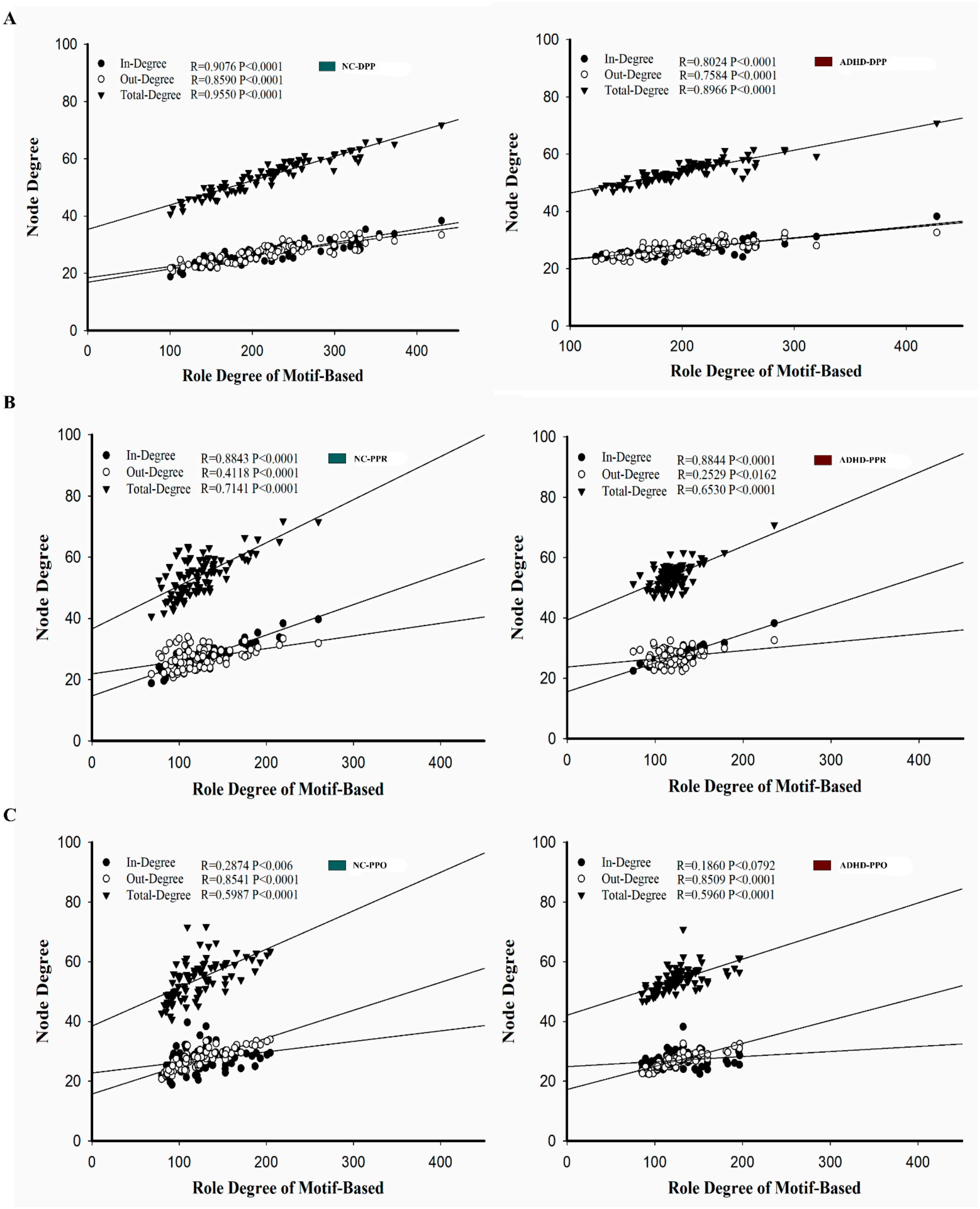

Supplementary Figure S3. The correlation between node degree(in-degree/out-degree) and motif-based role-degree.

3. Supplementary method

The calculation process of the model order p:

For the multivariate autoregressive (MVAR) model order p, values too small can lead to a poor representation of the data, whereas values too large can lead to problems of model estimation. Therefore, we used the Akaike information criterion (AIC) and

Bayesian information criterion (BIC) to select the optimal model order p. For n variables:

$$\text{AIC}\ (p) = \ln\left(\det(\Sigma)\right) + \frac{2pn^2}{\text{T}} \qquad \text{(S1)}$$

$$\text{BIC}\ (p) = \ln\left(\det(\Sigma)\right) + \frac{\ln(\text{T})pn^2}{\text{T}} \qquad \text{(S2)}$$

where Σ is the noise covariance matrix of the restricted model, and T is the time-series length. The results showed that, regardless of whether AIC or BIC was used, the error noise was the smallest when the model order p was set to 1.

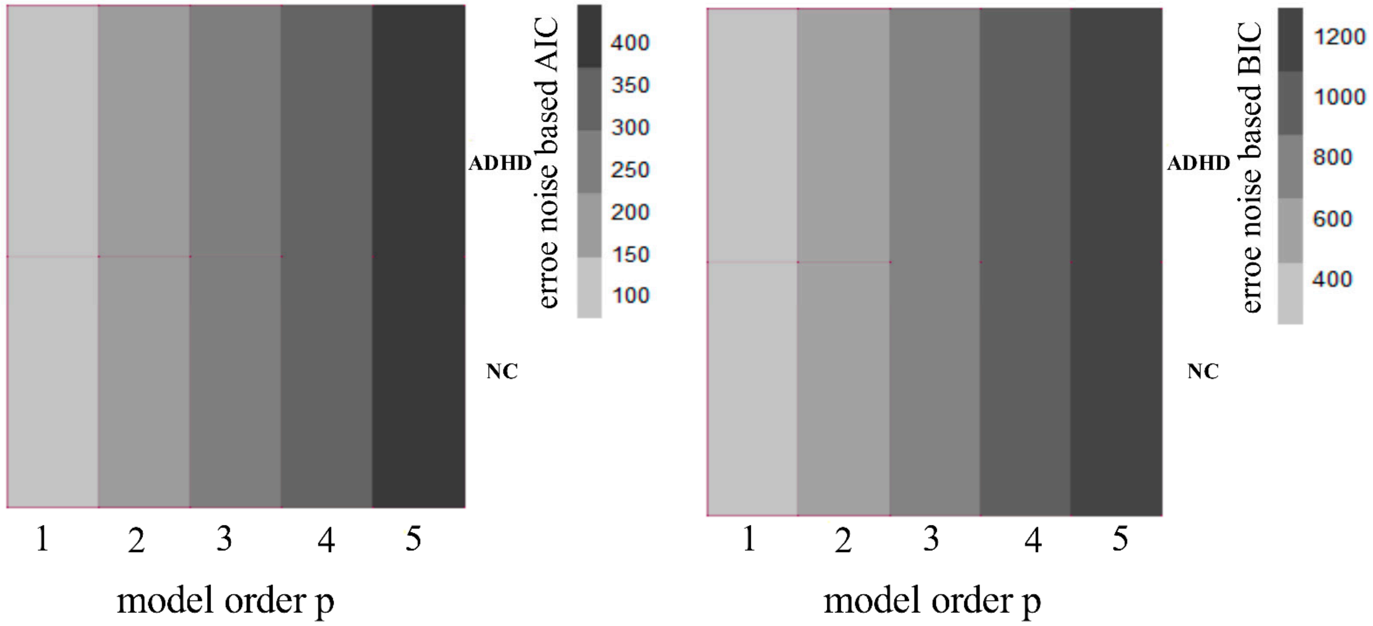

Supplementary Figure S4. The model order *p* of parameter optimization.
